# Creatine Supplementation in Depression: A Review of Mechanisms, Efficacy, Clinical Outcomes, and Future Directions

**DOI:** 10.7759/cureus.71638

**Published:** 2024-10-16

**Authors:** Keshav Juneja, Hamsa Priya Bhuchakra, Soumyodip Sadhukhan, Ishani Mehta, Alla Niharika, Swati Thareja, Tharun Nimmakayala, Sweta Sahu

**Affiliations:** 1 Psychiatry, Byramjee Jeejeebhoy (BJ) Medical College, Ahmedabad, IND; 2 Internal Medicine, Apollo Institute of Medical Sciences and Research, Hyderabad, IND; 3 Medical School, Dhaka National Medical College, Dhaka, BGD; 4 Psychiatry and Behavioral Sciences, Maharaja Agrasen Institute of Medical Research and Education, Hissar, IND; 5 Medical School, Sri Venkateswara Institute of Medical Sciences, Sri Padmavathi Medical College for Women, Tirupati, IND; 6 Medicine and Surgery, The Hans Foundation, New Delhi, IND; 7 Medicine and Surgery, Apollo Institute of Medical Sciences and Research, Chittoor, IND; 8 Internal Medicine, Jagadguru Jayadeva Murugarajendra (JJM) Medical College, Davangere, IND

**Keywords:** adjunctive therapy, brain energy metabolism, clinical trials, creatine supplementation, depression treatment, mental health, mood disorders, neuroprotection, ssri combination, treatment-resistant depression

## Abstract

Depression, affecting millions of people worldwide, is a leading cause of disability globally. It affects not only daily functioning but also interpersonal relationships and overall health by increasing the risks of chronic physical and mental illnesses. Creatine, traditionally recognized for boosting physical performance through its role in producing adenosine triphosphate, has recently shown potential as an adjunctive therapy for treating depression. Creatine’s ability to enhance brain energy metabolisms and provide neuroprotection suggests that it can alleviate mood disorders by improving mitochondrial function, increasing cellular resilience, and modulating neurotransmitter systems that regulate mood.

This narrative review aims to critically evaluate the research on creatine supplementation for depression, focusing on its efficacy, mechanism of action, risks, and benefits as a treatment for mood disorders. It analyzes preclinical and clinical studies to understand creatine’s potential as an adjunctive or alternative therapy for major depressive disorder and bipolar depression and underscores any gaps in current research. Both animal models and human trials indicate creatine’s efficacy for the treatment of depression. Creatine supplementation reduces depressive symptoms, particularly when combined with selective serotonin reuptake inhibitors, and may improve brain energy metabolism and neuroplasticity. It is generally well tolerated, though caution is warranted due to potential side effects such as manic episodes in bipolar disorder and renal function impairment in patients with kidney dysfunction. Overall, creatine presents a promising addition to current depression treatments, though further research is needed to establish optimal dosing, long-term efficacy, and safety across diverse patient populations.

## Introduction and background

Depression is a widespread mental health disorder that affects millions of people worldwide, significantly contributing to disability and reducing quality of life. According to the World Health Organization (WHO), depression ranks as one of the leading causes of disability, impacting around 280 million individuals globally and imposing a substantial economic and social burden [1]. People with depression frequently face a range of symptoms, including ongoing sadness, exhaustion, and trouble with thinking, which can significantly interfere with their daily lives. Although conventional treatments such as antidepressants, therapy, and lifestyle modifications are widely utilized, they do have their drawbacks. They may take time to work, may not be effective for everyone, and can sometimes cause unwanted side effects [2]. A large number of patients also suffer from treatment-resistant depression, where traditional therapies fail to provide sufficient relief, leading to an ongoing search for new, more effective treatments [3].

Creatine, a naturally occurring compound known for its role in energy metabolism, has long been used as a dietary supplement to boost physical performance. It plays a key role in producing adenosine triphosphate (ATP), the main energy source for cells, which is critical for maintaining optimal cellular function, especially during periods of high energy demand [4]. Although creatine is primarily associated with physical health and athletic performance, recent studies have expanded its relevance to brain function. Emerging evidence suggests that creatine may benefit brain energy metabolism, potentially improving cognitive processes, supporting neuronal survival, and influencing neurotransmitter function [5]. This has sparked growing interest in its potential as an add-on treatment for mood disorders, particularly depression.

The interest in creatine as a treatment for depression stems from its role in brain energy metabolism, which is often disrupted in individuals with depression. Mitochondrial dysfunction, reduced ATP production, and oxidative stress have all been linked to the development of depression, highlighting creatine’s potential neuroprotective properties [6]. Research has shown that creatine supplementation can enhance mitochondrial function, boost cellular resilience to stress, and affect key neurotransmitter systems, such as serotonin and dopamine, which are critical to mood regulation [7]. These mechanisms suggest that creatine might help reduce depressive symptoms by restoring energy balance in brain cells and protecting them from neuroinflammation and oxidative damage.

Recent clinical studies have started to explore creatine’s antidepressant potential. Both animal and human trials have shown early evidence of creatine’s positive effects on mood. In animal models of depression, creatine has been found to reverse depression-like behaviors, improve brain plasticity, and modulate key areas of the brain involved in mood regulation [8]. Human studies have also shown promising results, with some trials indicating that creatine can improve symptoms of major depressive disorder (MDD) and bipolar depression, especially when used alongside standard antidepressant treatments [9]. These findings offer hope that creatine could become a viable option for treating depression, particularly in cases where other treatments have failed, although more extensive research is still needed to confirm these results.

This narrative review aims to critically assess the current literature on creatine’s role in depression treatment, focusing on its underlying mechanisms, effectiveness, and safety. By bringing together evidence from both preclinical and clinical studies, this review seeks to provide a comprehensive understanding of creatine’s potential as a supplementary or alternative treatment for depression. Additionally, the review will address the limitations of the existing research and propose future directions for studying creatine’s role in mental healthcare.

## Review

Creatine: biological role and effect on brain function

Creatine is a naturally occurring compound found in meat and fish and synthesized endogenously by the liver, kidneys, and pancreas. It plays a crucial role in energy metabolism, serving as a rapid energy reserve in muscle and brain tissues. Skeletal muscle, which contains about 95% of the body’s creatine, depends on endogenous production or dietary intake for its creatine supply. In contrast, the brain can produce creatine independently due to the presence of key enzymes, i.e., arginine amidinotransferase, and S-adenosyl-l-methionine methyltransferase, in astrocytes, neurons, and oligodendrocytes [10].

**Figure 1:**
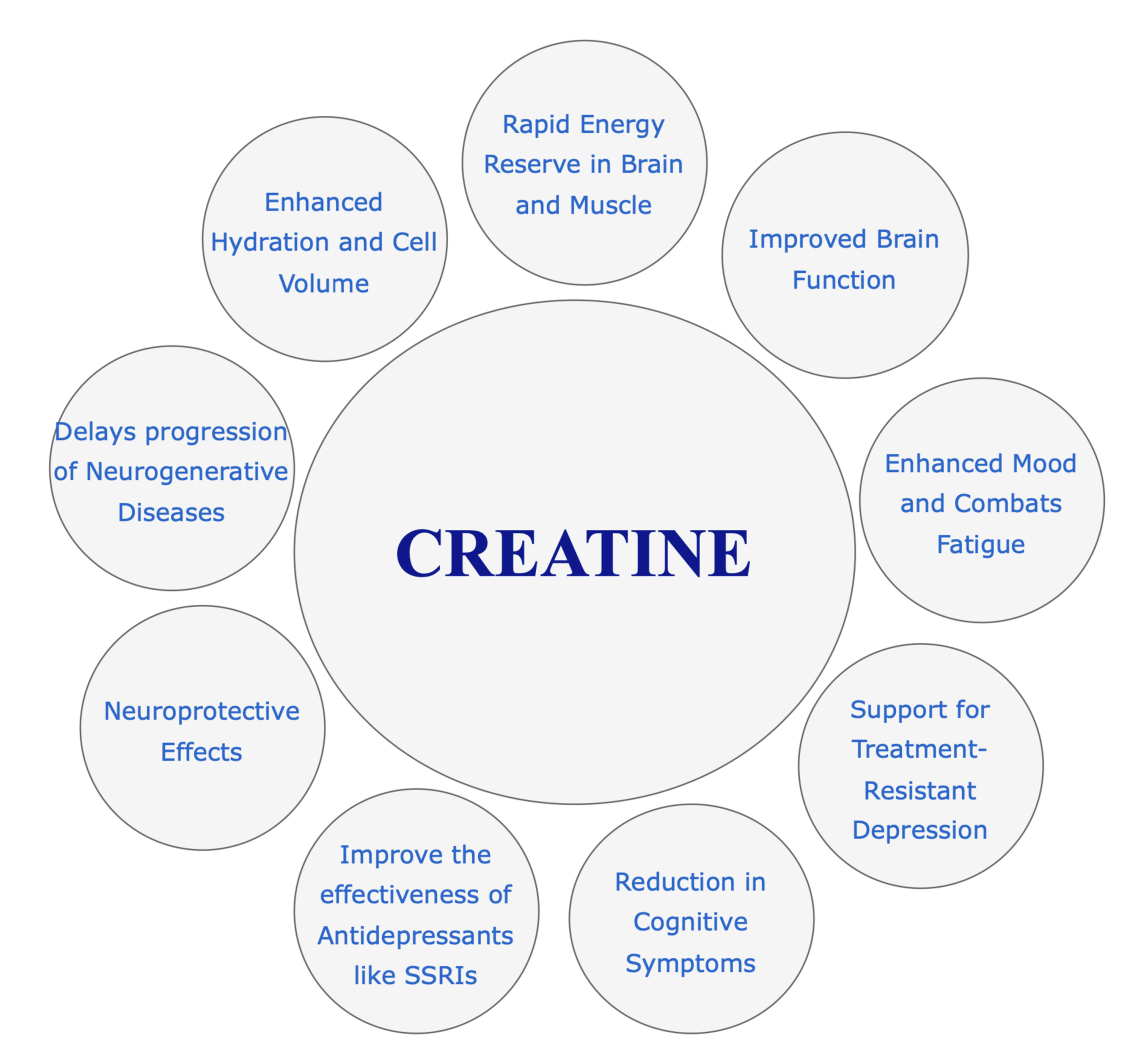
Creatine’s role and effect on brain functions. Image credits: Keshav Juneja, Hamsa Priya Bhuchakra, and Soumyodip Sadhukhan.

Vegetarians, with typically lower dietary creatine intake, show reduced muscle phosphocreatine levels compared to omnivores, yet there are no significant differences in brain phosphocreatine levels between the groups. This suggests that dietary intake does not have a significant impact on brain creatine levels [11]. Creatine gained popularity in the 1990s for enhancing resistance training, particularly in short, high-intensity exercises. Recent evidence suggests that creatine’s benefits extend beyond muscle health, potentially impacting brain function and offering therapeutic roles in neurological and psychiatric conditions, including depression. Although the ergogenic effects of creatine in muscles are well-established, its effects on brain function and optimal dosing strategies are still under investigation [4]. Figure 1 illustrates the overall effects of creatine on brain functions.

**Figure 2:**
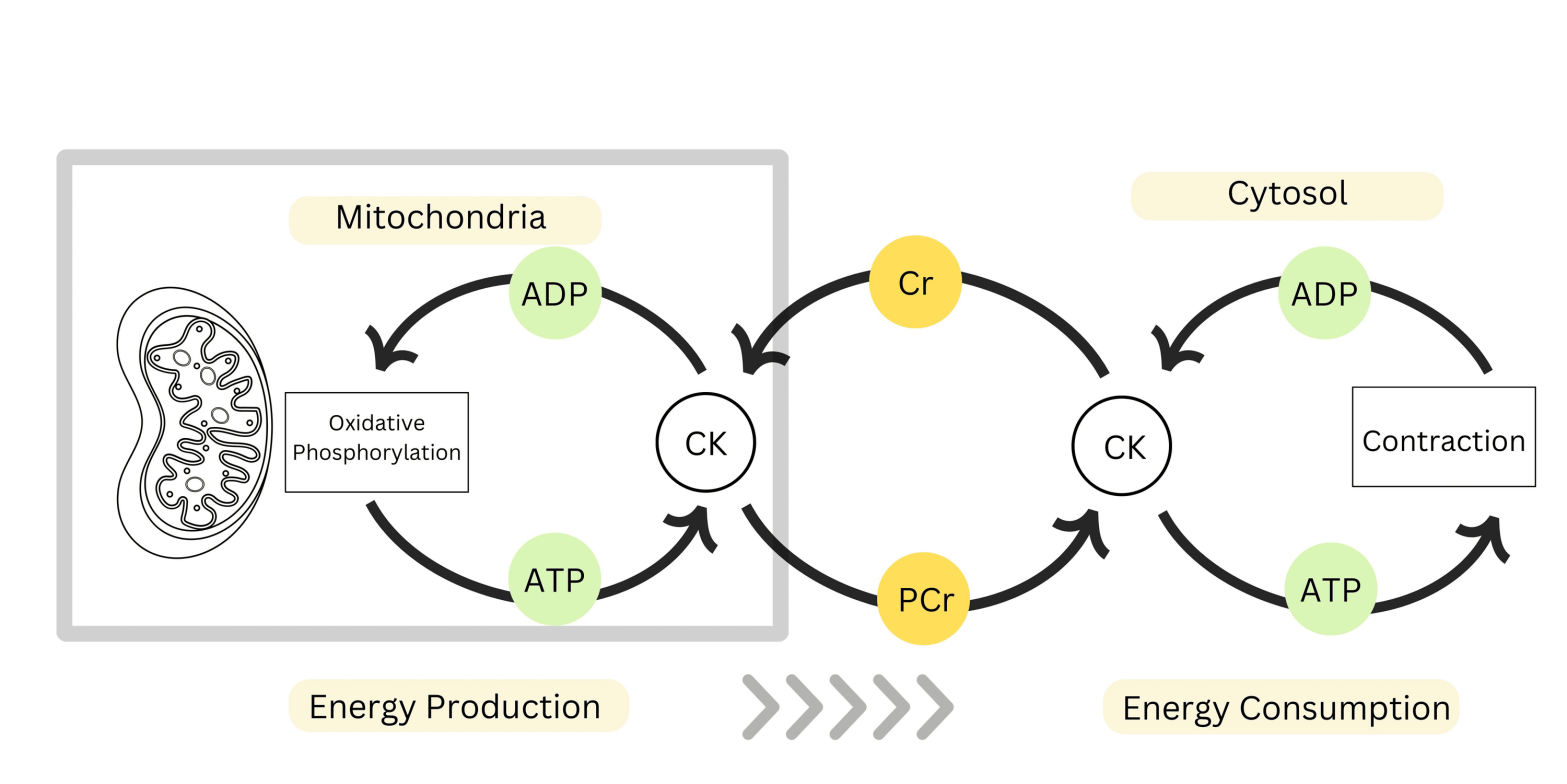
Phosphocreatine shuttle in skeletal muscle cells. The figure details the conversion of creatine (Cr) to phosphocreatine (Pcr) by creatine kinase (CK) and the regeneration of adenosine triphosphate (ATP) from adenosine diphosphate (ADP). Authors’ own image.

Creatine supplementation is particularly beneficial during periods of cognitive stress, such as sleep deprivation or complex cognitive tasks [12]. Preliminary evidence suggests it may aid recovery from mild traumatic brain injury, though human data are limited [13]. In skeletal muscle cells, creatine is transported and converted into phosphocreatine by the enzyme creatine kinase (CK). This reaction consumes ATP, converting it into adenosine diphosphate (ADP) in the process. Phosphocreatine acts as an energy reserve, as it donates a phosphate group to ADP to regenerate ATP when the muscle demands energy, especially during high-intensity, short-duration activities such as sprinting or weightlifting. The rapid regeneration of ATP by the phosphocreatine shuttle allows muscles to maintain energy levels without requiring immediate input from oxygen-dependent processes. This system primarily supports activities lasting less than 10 seconds, after which anaerobic glycolysis begins to contribute energy for slightly longer-duration efforts (Figure 2). Supplementing with creatine increases phosphocreatine stores, reducing muscle fatigue and enhancing performance in brief, high-intensity exercises and resistance training [14,15].

Creatine also plays a critical role in brain function due to the brain’s high energy demands. Although brain creatine constitutes less than 5% of total body creatine, it is essential for maintaining energy balance in metabolically active cells [9]. The brain relies on rapid ATP turnover, especially during complex cognitive tasks or high neural activity, with creatine facilitating ATP regeneration through the creatine phosphate system [16]. Evidence supports creatine’s importance, including the presence of CK isoforms in the central nervous system and associations between creatine deficiency and severe mental disorders, which can be partially mitigated by supplementation [17]. Recent studies suggest that creatine’s neuroprotective effects may also involve mechanisms beyond energy production, such as protecting against oxidative stress and regulating Na^+^/K+-ATPase and CAMKII/CREB [18]. These findings highlight the need for further research to understand creatine’s multifaceted impact on brain function and cognitive processing, with emerging evidence suggesting its potential as a therapeutic agent in conditions such as depression, schizophrenia, and after-brain injuries [19].

Research into creatine supplementation for cognitive enhancement has shown varied results. Generally, creatine seems to improve cognitive performance, particularly under stress conditions such as sleep deprivation and mental fatigue [20]. It may enhance cognitive function during hypoxia and combined stressors. However, results are inconsistent due to variations in study designs, including differences in stress conditions, dosages, and experimental protocols [21]. In older adults, the results are inconsistent; some studies show positive effects, while others find little to no improvement, which may be due to inadequate increases in brain creatine levels or the presence of neurodegenerative conditions [22]. Creatine supplementation may also benefit vegetarians more than meat-eaters due to lower baseline brain creatine levels, though the evidence is limited by methodological issues [23]. For athletes, creatine has been shown to mitigate mental fatigue in stressful contexts, though its effects in non-stressed conditions are less clear [24]. One recent study suggested potential cognitive benefits even without prior stressors [25], though further research is needed to clarify its role in specific sports and cognitive contexts.

Depression, affecting approximately 5% of adults globally, involves complex neurobiological pathways, including neurotransmitter imbalances, oxidative stress, and impaired energy metabolism. Disruptions in neurotransmitter systems, particularly serotonin, are central to depression. Oxidative stress, characterized by an imbalance between free radical production and antioxidant defenses, is significantly linked to depression. This condition exacerbates neuroinflammation and impairs neurogenesis and synaptic plasticity, which are crucial for cognitive function and emotional regulation. Understanding these pathways is essential for developing effective, personalized therapies for depression [26,27].

Creatine is emerging as a potential adjunctive treatment for depression due to its ability to counteract several neurobiological disturbances associated with the disorder. One key mechanism is its enhancement of cellular energy; by boosting ATP production, creatine helps restore energy balance and potentially alleviates depressive symptoms. Additionally, creatine’s neuroprotective properties may shield brain cells from damage caused by neuroinflammation and oxidative stress, which are prominent in depression. This protection may contribute to maintaining healthy brain function and improving mood. Creatine may also modulate neurotransmitter systems, such as serotonin and dopamine pathways, which are crucial for mood regulation. This modulation could enhance the effectiveness of conventional antidepressants. Moreover, creatine’s anti-inflammatory effects may help reduce inflammation associated with depression, leading to improved brain function and mood. Despite mixed clinical evidence, creatine supplementation shows promise as an adjunctive treatment for depression [28].

One recent study showed that biological sex, sexual hormones, and metabolic status mediate the effects of long-term creatine supplementation. Research indicates that combined creatine and sex hormone treatments can normalize neuroplasticity-related gene expression in gonadectomized rats, with notable antidepressant-like effects in females. However, creatine alone has shown negative effects on these factors in healthy rats, raising concerns about its clinical implications. These findings suggest that creatine supplementation may be most beneficial for individuals with significant metabolic impairments or elevated ATP demands, such as those observed in ovariectomized and gonadectomized rats. Future studies should explore interactions between creatine and variables such as sex, stress, exercise, drugs of abuse, prescription medications, and nutritional supplements. More basic neuroscience studies are needed to characterize creatine-induced changes in neurobiology and behavior, determine optimal dosing and time courses, and identify factors that influence the safety and effectiveness of creatine supplementation [29].

Clinical studies on creatine and depression

Animal Trials and Preclinical Studies

The research on the effects of creatine supplementation on mood-related behaviors, particularly concerning depression, has been expanding. Animal studies have provided valuable insights into creatine’s potential antidepressant properties and the underlying mechanisms.

A study by Allen et al. found that chronic creatine supplementation altered depression-like behavior in rodents in a sex-dependent manner, with female rodents showing more significant improvements compared to males. This highlights the importance of considering biological sex when examining creatine’s effects on mood [30]. Additionally, Ahn et al. investigated the combined effects of creatine supplementation and exercise in mice, demonstrating that this combination improved depressive-like behaviors and enhanced serotonin (5-HT) neuron activity in the raphe nuclei. This suggests that creatine, especially when paired with exercise, may positively influence serotonergic pathways involved in mood regulation [31].

Further exploring creatine’s antidepressant properties, Cunha et al. showed that creatine, similar to ketamine, produces antidepressant-like effects in a mouse model of depression. These effects were found to involve the activation of adenosine A1 and A2A receptors, providing a mechanistic explanation for creatine’s mood-enhancing effects beyond the traditional serotonergic pathways [32]. Kanekar et al. expanded on the sex-dependent impact of creatine supplementation in an animal model of treatment-resistant depression, finding that creatine not only reduced depressive symptoms but also enhanced the efficacy of selective serotonin reuptake inhibitors (SSRIs) in female animals. This highlights creatine’s potential as an adjunctive treatment to improve the therapeutic response in individuals resistant to conventional antidepressant therapies [33].

Additionally, Leem et al. demonstrated that regular exercise combined with creatine supplementation could prevent stress-induced decreases in hippocampal neurogenesis via the Wnt/GSK3β/β-catenin pathway, suggesting that creatine may protect against the neurobiological changes associated with chronic stress and depression [34]. Furthermore, Kim et al. found that creatine, in combination with taurine, alleviated depressive-like behavior in both *Drosophila* and mice by regulating the protein kinase B (Akt) and extracellular signal-regulated kinase/brain-derived neurotrophic factor pathways, further supporting creatine’s role in enhancing neuroplasticity and mood regulation [35].

These studies indicate that creatine may exert antidepressant effects through multiple pathways, including serotonin modulation, adenosine receptor activation, and neurogenesis. These findings pave the way for future research into creatine as a therapeutic strategy for depression.

Human Trials

Creatine, a compound primarily known for its role in muscle energy metabolism, has garnered attention for its potential antidepressant effects. Recent human trials have explored its efficacy in treating MDD and bipolar depression, examining various dosages, treatment durations, and patient populations. This summary highlights the key findings from these studies, including efficacy, safety, and side effects.

A large-scale observational study by Bakian et al. investigated the relationship between dietary creatine intake and depression risk among U.S. adults. The findings suggested an inverse association, indicating that higher creatine consumption may be beneficial for mood regulation (adjusted odds ratio = 0.68, 95% confidence interval = 0.52-0.88) [36]. Additionally, research on creatine’s impact on brain function and health has been promising. Studies have reviewed creatine’s potential benefits in supporting cognitive processes and potentially alleviating symptoms of depression [37,38]. New research has shed light on the potential mechanisms by which creatine works as an antidepressant. Kondo et al. studied the effects of creatine on brain bioenergetics in adolescents with SSRI-resistant depression using phosphorus-31 magnetic resonance spectroscopy and discovered that elevated brain phosphocreatine levels were linked to better mood [39]. The biochemical and neuroimaging properties of creatine have also been studied by Chen et al. and Tran et al. These investigations have shown modifications in brain energy metabolism and neurotransmitter systems, which may be the basis for creatine’s antidepressant effects [40,41]. Furthermore, another study examined creatine levels and metabolites containing choline in connection to the recurrence of depressive episodes, and the results suggested that creatine might have an impact on metabolic biomarkers associated with depression [42]. Creatine monohydrate has been investigated in clinical studies as an additional therapy for bipolar depression. When combined with normal therapies, creatine supplementation decreased depressive symptoms in people with bipolar depression, according to a randomized, double-blind, placebo-controlled experiment [43].

Further investigation examined creatine’s function in combination treatments. Potential advantages were shown in open-label pilot research that assessed the combination use of 5-hydroxytryptophan and creatine monohydrate for adult women with serotonin-norepinephrine reuptake inhibitor (SNRI)-resistant depression [44]. Likewise, a different study found that by altering brain bioenergetics, creatine may improve the effectiveness of SSRIs in treating treatment-resistant depression [39,44].

Researchers in clinical trials for creatine have used different dosages and treatment durations of three to eight weeks, typically ranging from 3 to 10 g daily. For instance, Kious et al. studied the effects of giving MDD patients 5 g of creatine each day together with SSRIs for eight weeks. In comparison to a placebo, this study’s goal was to increase the antidepressant benefits of SSRIs. The study found significant improvements in depressed symptoms [45]. Similarly, Toniolo et al. focused on the medication’s potential as an additional treatment by utilizing a larger dose of 6 g/day for six weeks in individuals with bipolar depression [43]. In contrast, Chen et al.’s pilot trial investigating the intravenous administration of phosphocreatine and ATP in combination with fluoxetine demonstrated a creative method of incorporating creatine into treatment plans. While preliminary, this technique appears to have the potential to improve therapy outcomes [40].

Efficacy

Creatine’s efficacy in treating depression has been generally beneficial; however, the results vary by study. Adults with MDD who were either unmedicated or not responding to SSRIs participated in a randomized controlled trial by Kious et al. Their findings revealed that creatine supplementation considerably alleviated depression symptoms compared to placebo [28,44]. Toniolo et al., on the other hand, investigated creatine in patients with bipolar depression and discovered that it relieved depressed symptoms in bipolar patients, but it had no meaningful impact on cognitive assessments and raised concerns about probable manic episodes [43]. A total of 52 female patients with unipolar depression participated in a double-blind, placebo-controlled study by Lyoo et al., which demonstrated that adding creatine monohydrate (5 g daily) as a safe and useful supplement to escitalopram treatment for eight weeks was successful. Additionally, they discovered a noteworthy antidepressant effect: the creatine group’s Hamilton Depression Rating Scale scores decreased by 79.7%, while the placebo group’s scores decreased by 62.5% [9].

Safety and Side Effects

Most investigations have shown that creatine is well tolerated. However, there are issues with bipolar depression. Toniolo et al. pointed out that some patients had manic episodes while taking mood stabilizers, which emphasizes the need for caution when using these medications in this population [28,43]. As we hypothesize that bipolar disorder may consist of a biphasic disorder of energy generation, increased mania, and decreased depression, we could reasonably expect that boosting mitochondrial energy generation may trigger manic symptoms, even though no causal role for creatine in these events can be established [44]. According to Kious et al., there was no discernible rise in side effects compared to the placebo, indicating a good safety profile [46]. Supplementing with creatine typically has modest side effects. Creatine was well tolerated in Hellem et al.’s trial of women with methamphetamine dependency, with no serious side effects reported [46]. On the other hand, Toniolo et al.’s observation that bipolar depression patients may experience manic episodes implies that careful observation is necessary [43].

Overall, the research suggests that creatine supplementation can be a promising supplementary treatment for depression, particularly in MDD, and may be beneficial for bipolar depression [47]. To improve treatment procedures, comprehend underlying mechanisms, and create long-term safety profiles, further investigation is necessary. To better leverage creatine’s potential advantages, future research should focus on determining effective dosages, treatment durations, and the effects of creatine on various mood disorders.

Creatine as an adjunctive therapy

Studies Evaluating Creatine as a Supplement to Traditional Antidepressant Treatments

Creatine is a natural compound found in the body that helps provide energy for muscles. It is often used as a supplement by athletes to improve muscle performance. However, recent research has suggested that creatine may also have benefits for mental health, particularly in individuals with depression. SSRIs are a common type of antidepressant medication. They work by increasing levels of serotonin, a neurotransmitter that plays a crucial role in mood regulation. A randomized, placebo-controlled trial was conducted in South Korea to investigate the effects of creatine supplementation as an adjunct to an SSRI in women with MDD. The group receiving creatine demonstrated a more rapid onset of antidepressant effects, as early as week two, and increased overall efficacy compared to the placebo group [9]. This suggests that creatine may enhance the effectiveness of SSRI treatment.

The Forced Swim Test (FST) is a widely used animal model to assess depressive-like behaviors. In this test, rodents are placed in a small, inescapable tank of water, and their immobility time is measured. Prolonged immobility is often interpreted as a sign of despair or hopelessness, similar to the symptoms experienced by individuals with depression. Creatine supplementation in the FST has been shown to reduce immobility time in female rodents, suggesting that creatine may have antidepressant-like effects in female rodents [30]. Phosphocreatine is a high-energy phosphate compound found in muscle cells. It plays a crucial role in providing energy for cellular processes. In the context of brain function, phosphocreatine levels are indicative of energy metabolism and overall brain health. The frontal lobe phosphocreatine was inversely correlated with depression scores (p = 0.02) in a randomized control trial where a magnetic resonance spectroscopy study of adolescent females with SSRI-resistant depression was done [39]. This means that as phosphocreatine levels in the frontal lobe increased, depression scores tended to decrease.

Magnetic resonance spectroscopy, a non-invasive brain imaging technique, measures brain metabolite levels. Studies show that depression is often linked to altered brain energy metabolism, with lower levels of phosphocreatine and ATP. Creatine supplements might help improve this and potentially alleviate depression symptoms [48].

Mechanism of Potential Synergistic Effects

Oral intake of creatine increases cerebral phosphocreatine and modifies brain bioenergetics by influencing high-energy phosphate metabolism, which results in increased ATP levels [49]. Brain adenosine levels have antidepressant effects. Creatine can acutely reverse the corticosterone-induced depressive-like behavior by a mechanism dependent on the PI3K/AKT/mTOR (PAM) pathway [50]. The antidepressant-like effect of creatine is dependent on PKA, CaMK-II, PKC, and MEK 1/2 activation [51].

When combined with SSRIs, creatine supplementation has been shown to accelerate the onset of antidepressant effects. Hence, individuals taking both creatine and an SSRI may experience a more rapid improvement in their mood symptoms compared to those taking only an SSRI [9]. 5-HT1A receptors are serotonin receptors involved in mood regulation. Creatine might have antidepressant effects by interacting with these receptors [52].

Excessive glutamate can damage brain cells (excitotoxicity) and is linked to depression and neurodegenerative diseases. Creatine, with antioxidant properties, protects brain cells from this damage. It can improve cognitive function and enhance the effectiveness of other treatments [53].

Clinical Outcomes and Challenges in Combined Therapy

Therapies that may be beneficial for treatment-refractory depression, such as electroconvulsive therapy or ketamine, are not widely available [54]. Creatine, being a popular nutritional supplement, is widely available. Antidepressants may produce some improvement within the first week or two of use yet full benefits may not be seen for two to three months; thus, adjuvant therapy becomes more beneficial as it shows a rapid response [9,55]. The remission rate at the endpoint was higher in the creatine group than in the placebo group in a randomized, placebo-controlled trial [9]. Creatine supplements did not help women with depression not responding to SSRIs, SNRIs, or noradrenergic and specific serotonergic antidepressants [56]. Creatine supplementation alters depression-like behavior in the FST in a sex-dependent manner in rodents [30]. Creatine supplements in people with a history of renal diseases, gastrointestinal disturbances, or non-steroidal anti-inflammatory drug use are to be practiced only after a proper medical consultation [57].

Clinical implications and practical considerations

In recent years, the clinical implications of prescribing creatine for depression have become increasingly relevant due to its promising role as an adjunct to traditional antidepressants. It has been hypothesized that creatine supplementation enhances the antidepressant response when administered with SSRIs. This process is especially evident in female and adolescent patients [39,58,59]. Creatine’s role in improving brain energy metabolism, a process often disrupted in depression, provides a new therapeutic avenue for patients [60].

The standard dosage used in clinical trials for depression is 4 to 5 g/day of creatine monohydrate, taken orally, usually in powder or capsule form, and can be mixed with water or other beverages. This is similar to the dose used in athletic performance studies. Studies suggest that improvements in depressive symptoms can occur within two to eight weeks of supplementation, particularly when used in conjunction with antidepressants such as SSRIs [61].

When administering creatine as part of treatment for depression, several patient characteristics are considered to ensure it is both safe and effective. For instance, patients with low dietary creatine intake, such as vegetarians or vegans and those with regular physical activity, may respond more favorably to supplementation because they generally have lower baseline levels [62]. Alcohol or drug consumption can affect how the body processes supplements such as creatine, and may also interact with antidepressant medications [63]. Some studies suggest that younger individuals and women might respond better to creatine supplementation, particularly in treatment-resistant depression [39,58,59]. On the other hand, it should be avoided in patients with hepatic disease or hypertension [61]. Notably, creatine can provoke manic/hypomanic switches in depressed bipolar patients [8].

As creatine is processed by the kidneys, patients with impaired renal function or taking medications that may disrupt it (e.g., diuretics or nephrotoxic drugs) need careful monitoring to avoid complications [63]. Similarly, patients who are already on medications for depression may need to be monitored for potential interactions or enhanced effects [39].

It is to be noted that different doses should be considered in patients with different muscle mass, as creatine can lead to water retention in muscles; therefore, individuals with higher muscle mass might metabolize creatine differently compared to others [64].

Generally, mild side effects are reported with creatine use, for example, gastrointestinal discomfort (e.g., bloating or diarrhea), water retention, tension headache, nausea and/or vomiting, and sleep difficulties [58,65,66]. Overall, it is well-tolerated and safe when used at appropriate doses. This makes it a valuable option for clinicians managing treatment-resistant depression, provided that it is administered carefully and tailored to the individual’s specific health profile.

Limitations and confounding factors in current research

The relationship between creatine supplementation and its effect on depression is complex, and, as a particular research area of interest, it has several limitations and confounding factors [67]. These include methodological challenges, dosage and duration of creatine supplementation, and individual variability.

The limited number of randomized control trials compared to correlational or observational studies and a smaller sample size resulting from methodological constraints impact the generalizability of the findings. Furthermore, it is essential to include a diverse population to comprehend the possible impacts of creatine supplementation on depression across various demographic groups.

Being an ongoing area of research, it is also difficult to determine the optimal dosage of creatine required for its potential antidepressant effects. Moreover, different dosages may be required for different demographics to respond in a certain way. The duration of creatine supplementation needed to create a potential antidepressant effect is also uncertain. Therefore, to determine the optimum dosage and duration, long-term studies are needed to assess the potential benefits and risks of creatine supplementation. Individual variability, including individual genetic factors, also plays a great role in influencing individual responses to creatine supplementation. Some individuals may be less or more susceptible to the antidepressant effects of creatine. In other cases, the co-existing mental health or medical conditions can confound the effect of creatine supplementation on depression.

Confounding factors also play an important role in such studies. These factors can introduce bias or affect the true relationship between the effects of creatine supplementation and depression. Creatine supplementation and depression studies mostly involve individuals who engage in regular exercise routines, making it difficult to separate the known benefits of exercise on mental health from those of creatine supplementation. It has been proven in various studies that exercise alone can alleviate the symptoms of depression [68]. Along with exercise, regular intake of a well-balanced diet is also known to lead to optimal brain function and mood regulation, thereby leading to a bias [69].

Some patients use creatine along with other antidepressants, and the use of other medications, mainly SNRIs and SSRIs, along with creatine supplementation induces a bias and can complicate the relationship between creatine supplementation and the alleviation of depressive symptoms. Many medications can also have drug-drug interactions with creatine, further complicating the outcomes of this study [70].

The placebo effect can also introduce bias and complicate the relationship between creatine supplementation and depression. The placebo effect occurs when the patient experiences positive outcomes due to their belief in the treatment. Apart from this, a good lifestyle, genetic factors, and underlying medical conditions, including mental health conditions, can also influence the individual response to creatine supplementation.

To address these limitations and confounding factors, future research must focus on well-designed randomized controlled trials, diverse populations, larger sample sizes, and careful control of the biases.

Future directions for research

From the above discussion, it can be well established that creatine supplementation has favorable antidepressant effects; however, research is lacking or inconsistent regarding the efficacy of creatine. All the data presented in this review are derived from either preclinical research or a limited number of small-scale human trials. Such a condition demands larger-scale clinical or randomized control trials worldwide, which need to include quantification of brain creatine and dietary measures to better understand habitual or supplemented dietary intake of creatine in response to such an intervention. This may help explain some of the preliminary benefits of creatine supplementation on indices of cognition and depression [71], among others.

It is important to determine the optimal creatine protocol capable of increasing brain creatine levels. To date, dose-response studies are lacking and protocols are heterogeneous [72]. Studies in which the potential supplementary benefits of creatine are assessed and concomitant assessment of brain creatine levels along with cognitive function may be involved, as it would establish the effect of creatine supplementation. The identification of novel conditions in which creatine supplementation may be as effective as creatine in a rested, healthy brain has been shown to have a reduced effect on cognition. Trials can be brought to establish creatine as a monotherapy and/or examine the potential efficacy of creatine as an augmenting agent when combined with neurostimulatory techniques for better characterization of the neurochemical and network-level effects of creatine and their correlations with antidepressant responses. Such evaluations are required to proceed further to deduce creatine response-specific subpopulations such as treatment-resistant depression or other mood disorders.

The efficacy of creatine for treating symptoms of depression must be evaluated along with clinical trials examining the effects of creatine (independent of pharmacological interventions) on this mood disorder, which are needed in greater numbers before a consensus can be reached. Future research is needed to determine the mechanistic effects of long-term creatine supplementation dosing strategies, with and without exercise, on brain function and health. Furthermore, it must be determined whether there are sex and age-related differences in response to creatine supplementation. Future multifactorial interventions may involve creatine combined with other strategies to enhance patient outcomes.

## Conclusions

This review highlights that creatine, traditionally used to enhance physical performance, shows promise as an adjunctive management option for depression. Research shows that creatine has a multifaceted mechanism, boosting brain energy metabolism, modulating neurotransmitter systems, and providing neuroprotection, especially when combined with antidepressants at a dose of 4-5 g/day for two to eight weeks. While current research highlights significant promise, particularly among female and adolescent patients with treatment-resistant depression and those with low baseline creatine levels, creatine should be used with caution in patients with renal dysfunction and bipolar disorder. However, there are limitations to current research, including small sample sizes, heterogeneous study designs, inconsistent methodologies, and a lack of dose-response trials. The lack of conclusion on its long-term efficacy, impact on diverse populations, optimal dosing regimens, and potential benefits calls for robust research. Further large-scale trials are required to fully understand and realize creatine’s efficacy as a treatment for depression.
